# Vitamin D Resistance as a Possible Cause of Autoimmune Diseases: A Hypothesis Confirmed by a Therapeutic High-Dose Vitamin D Protocol

**DOI:** 10.3389/fimmu.2021.655739

**Published:** 2021-04-07

**Authors:** Dirk Lemke, Rainer Johannes Klement, Felix Schweiger, Beatrix Schweiger, Jörg Spitz

**Affiliations:** ^1^Praxis Dr. Beatrix Schweiger, Bensheim, Germany; ^2^Department of Radiotherapy and Radiation Oncology, Leopoldina Hospital Schweinfurt, Schweinfurt, Germany; ^3^Praxis Dr. Beatrix Schweiger, Waldkirch, Germany; ^4^Akademie für menschliche Medizin und evolutionäre Gesundheit, Schlangenbad, Germany

**Keywords:** autoimmune diseases, Coimbra protocol, multiple sclerosis, vitamin D receptor (VDR), vitamin D

## Abstract

Vitamin D_3_ (cholecalciferol) is a secosteroid and prohormone which is metabolized in various tissues to the biologically most active vitamin D hormone 1,25(OH)_2_D_3_ (calcitriol). 1,25(OH)_2_D_3_ has multiple pleiotropic effects, particularly within the immune system, and is increasingly utilized not only within prophylaxis, but also within therapy of various diseases. In this context, the latest research has revealed clinical benefits of high dose vitamin D_3_ therapy in autoimmune diseases. The necessity of high doses of vitamin D_3_ for treatment success can be explained by the concept of an acquired form of vitamin D resistance. Its etiology is based on the one hand on polymorphisms within genes affecting the vitamin D system, causing susceptibility towards developing low vitamin D responsiveness and autoimmune diseases; on the other hand it is based on a blockade of vitamin D receptor signaling, e.g. through pathogen infections. In this paper, we review observational and mechanistic evidence for the acquired vitamin D resistance hypothesis. We particularly focus on its clinical confirmation from our experience of treating multiple sclerosis patients with the so-called Coimbra protocol, in which daily doses up to 1000 I.U. vitamin D_3_ per kg body weight can be administered safely. Parathyroid hormone levels in serum thereby provide the key information for finding the right dose. We argue that acquired vitamin D resistance provides a plausible pathomechanism for the development of autoimmune diseases, which could be treated using high-dose vitamin D_3_ therapy.

## Introduction

Vitamin D is a secosteroid and prohormone which can be obtained from food (as either vitamin D_2_ or D_3_), but whose main source is endogenous production in the skin. This requires ultraviolet-B radiation (290-315 nm) of at least 18 mJ/cm^2^ intensity (1), inducing the formation of previtamin D_3_ from 7-dehydrocholesterol which subsequently converts to vitamin D_3_ (cholecalciferol) by body heat (2). Upon reaching the blood, vitamin D_3_ mainly binds to vitamin-D binding protein (DBP) and gets transported to the liver where it is transformed into its storage form calcidiol (25-hydroxyvitamin D_3_ or 25(OH)D_3_) through hydroxylation *via* the vitamin D_3_ 25-hydroxylases CYP2R1, CYP27A1 or CYP27B1, all members of the cytochrome P450 enzyme family (3, 4). 25(OH)D_3_ is the main laboratory parameter to judge an individual’s vitamin D status; concentrations <20 ng/ml (1ng/ml = 2,5 nmol/l) are considered as vitamin D deficiency, and 40-60 ng/ml as ideal (2, 5). 25(OH)D_3_ is metabolized in various tissues (predominantly the kidneys) to the biologically most active vitamin D hormone calcitriol (1,25-dihydroxyvitamin D or 1,25(OH)_2_D_3_) with another hydroxylation at the 1α position by CYP27A1 or CYP27B1 (3, 4). Besides regulating calcium metabolism, 1,25(OH)_2_D_3_ has multiple pleiotropic effects, particularly within the immune system, and is increasingly utilized not only within prophylaxis, but also within therapy of various diseases (2, 6, 7). In particular, binding of 1,25(OH)_2_D_3_ to the vitamin D receptor (VDR) has been shown to inhibit the differentiation and proliferation of B and T helper (Th) lymphocytes, promoting the shift of an inflammatory to a more tolerant immune status which may explain the protective effects of vitamin D against autoimmune diseases [reviewed in (8)].

More recently, Slominski and colleagues have revealed alternative pathways of vitamin D metabolism mediated by the mitochondrial enzyme CYP11A1 which is able to hydroxylate the side chain of vitamin D_2_/D_3_ (9–11). The main product of these reactions is 20-hydroxyvitamin D_3_ (20(OH)D_3_), which has a 20-30-fold lower concentration than 25(OH)D_3_ in human serum (9, 11) and is the initial substrate for the formation of further hydroxy-derivatives such as 20,23(OH)_2_D_3_, 17,20,23(OH)_3_D_3_ or 20,22(OH)_2_D_3_ [reviewed in (12)]. These nonclassical vitamin D metabolites also act as hormones: besides being partial agonists of the VDR, they have high affinity as agonists of the aryl hydrocarbon receptor (AhR) (13) and as inverse agonists of the retinoid-related orphan receptors (ROR) α and γ (14, 15). Notably, CYP11A1 is expressed in immune cells (16) and RORα and RORγ are expressed by inflammatory Th17 cells in which they synergistically regulate differentiation and inflammatory cytokine production (17). Th17-derived cytokines, notably interleukin (IL)17, have been implicated in the etiology of autoimmune disorders such as psoriasis (18) and multiple sclerosis (MS) (19). In addition, single nucleotide polymorphisms (SNPs) of the *RORA* gene have been associated with MS (20). The binding of 1,25(OH)_2_D_3_, 20(OH)D and other vitamin D hydroxy-metabolites to both RORα and RORγ, result in IL17 inhibition (14), thus providing another mechanism distinct from VDR signaling how vitamin D may protect from, or alleviate symptoms of, autoimmune diseases.

For approximately 15 years, patients with autoimmune diseases, particularly MS, have been successfully treated using a high-dose vitamin D protocol. Because this method has been developed by Prof. Dr. Cicero Coimbra in Sao Paolo, Brazil, it is frequently referred to as the “Coimbra protocol”; in Germany it is utilized since 2016. Underlying the Coimbra protocol is the hypothesis of a non-hereditary, but acquired form of vitamin D resistance which this paper is going to examine.

A hallmark of acquired vitamin D resistance, if it exists, would be an elevated parathyroid hormone (PTH) concentration despite 25(OH)D_3_ levels being in the ideal range and thus indicating sufficient production of 1,25(OH)_2_D_3_. One key role of 1,25(OH)_2_D_3_ is to enhance intestinal calcium absorption. If ionized calcium concentrations in blood are low, the parathyroid glands release PTH which stimulates calcium release from bones. Furthermore, PTH increases the conversion of 25(OH)D_3_ into 1,25(OH)_2_D_3_ in the kidneys with subsequent release into circulation. PTH also inhibits the tubular reabsorption of phosphate which in turn lowers the amount of water-insoluble calcium-phosphate salts and thus increases ionized calcium concentrations. In this way, PTH constitutes a direct feedback mechanism within the vitamin D system. A physiological 25(OH)D_3_ level should thereby be able to suppress PTH into the lower third of the reference range. In other words: If 25(OH)D_3_ levels are high, PTH should be low and vice versa. In patients with autoimmune diseases this negative feedback loop is disturbed. Based on these observations, Prof. Coimbra proposed the hypothesis of a vitamin D resistance.

## The Hypothesis of Acquired Vitamin D Resistance

In two intervention studies, Carlberg and colleagues found evidence that different individuals display a different molecular and biochemical response to the same dose of either long-term or single-bolus vitamin D_3_ supplementation (21). Initially, in the VitDmet study, 71 elderly prediabetic individuals were supplemented with either 0, 1600 or 3200 I.U. vitamin D_3_ daily over 5 months of Finnish winter (22). The focus of this work was on the effects of vitamin D_3_ supplementation on mRNA expression of twelve of the vitamin D-regulated genes and several vitamin D-affected laboratory parameters. With the help of these biomarkers, the group was able to show in 2015 that even supposedly adequate high vitamin D_3_ doses (3200 I.U.) were not able to exert the expected vitamin D-regulatory effects in all subjects. Focusing on the PTH feedback system alone, a total of 25% of the patients showed no adequate response of this laboratory parameter. Regarding all 36 tested parameters, Carlberg et al. could cluster their patients in 24% low responders, 51% mid responders and 25% high responders. In 2017, the group was able to reproduce these findings in the VitDbol study, in which a cohort of healthy Finnish students received a 80,000 I.U. bolus dose of vitamin D_3_ with similar low responder rates (23).

These data provided an *in vivo* confirmation that there exists a spectrum of different vitamin D responsiveness, with approximately 25% of a population not responding adequately to conventional vitamin D_3_ doses. They may require individually varying, but larger doses. Vitamin D resistance, as proposed by Prof. Coimbra and this article, could be conceived as the extreme low-response end of the vitamin D responsiveness spectrum. Accordingly, individuals being vitamin D resistant would require very high doses of vitamin D_3_ supplementation to achieve an adequate physiological response, such as a reduction of PTH concentrations or the down-regulation of an activated adaptive immune system – the latter effect is important for the treatment of autoimmune diseases as discussed further below.

Indeed, the idea of vitamin D resistance has first been proposed in 1937 by Albright, Butler and Bloomberg based on the observation that in rare cases of rickets in children, very high doses of vitamin D were required to relieve symptoms (24). It has later been shown that resistance to 1,25(OH)_2_D_3_ in such children is frequently caused by hereditary VDR defects, resulting in hypocalcemia, secondary hyperparathyroidism, rickets and in about one half of the patients alopecia (25–27). In contrast to these cases of hereditary vitamin D resistance, which is very rare and already diagnosed in childhood, the hypothesis investigated in this paper concerns a non-hereditary, acquired form of vitamin D resistance which promotes the development of autoimmune diseases. As discussed in more detail below, such a form of vitamin D resistance could develop during aging based on an interaction between genetic susceptibility polymorphisms of the vitamin D system and an accumulation of environmental factors additionally impairing the hormonal signaling of the vitamin D-derived hydroxy-metabolites. Acquired vitamin D resistance is therefore more common than hereditary vitamin D resistance according to the frequency of susceptibility polymorphisms and the rising incidence of autoimmune diseases.

## Diagnosis of Vitamin D Resistance

In their article “The concept of the personal vitamin D response index”, Carlberg and Haq (21) proposed the following: “A screening using a single vitamin D bolus treatment and measurements at days 0 and 2 paired with a simplified protocol of the molecular analysis used in the VitDbol study [… ] may be the easiest way to identify persons with a low vitamin D response index” (21). Theoretically, people with acquired vitamin D resistance should also be found with this protocol as outliers with extremely low response indices. However, the majority of the biomarkers measured in the VitDbol study can only be determined in research laboratories. Nevertheless, PTH provides a valuable and easy-to-measure biomarker for the clinical therapist. As mentioned above 1,25(OH)_2_D_3_ and PTH stand in a direct relationship with each other. A high PTH concentration in turn is associated with a higher all-cause-mortality in epidemiological studies (28). Hence, the recommended optimal 25(OH)D_3_ level of >40ng/ml should correlate physiologically with an optimal PTH concentration. Because reference ranges and measurement units for PTH vary among different laboratories, it could be stated that a 25(OH)D_3_ measurement >40 ng/ml should be associated with a PTH value in the middle of the lower third of its laboratory-specific reference range. In such a case, high PTH values are indicative for vitamin D resistance, assuming that dietary calcium and phosphate intake are adequate and a differential diagnosis of hyperparathyroidism has been ruled out. For example, if a laboratory’s reference range for PTH is 15-65 ng/ml, the middle of the lower third of that range would be 23.3 ng/ml.

Furthermore, the constellation of high serum 1,25(OH)_2_D_3_ concentrations with a concurrently physiological 25(OH)D_3_ concentration is known to occur in hereditary forms of vitamin D resistance or VDR knockout mice (29); it could therefore also be indicative of acquired vitamin D resistance. The 1,25(OH)_2_D_3_ concentration is determined on the one hand by the activity of the enzymes CYP2R1, CYP27A1 and CYP27B1 that catalyze production of 1,25(OH)_2_D_3_ and on the other hand CYP24A1 which catalyzes 1,25(OH)_2_D_3_ degradation. The inactivation of 1,25(OH)_2_D_3_ occurs either systemically or locally within the cells of target tissues. Because PTH negatively regulates CYP24A1 and positively regulates the hydroxylases converting 25(OH)D_3_ to 1,25(OH)_2_D_3_, the presence of vitamin D resistance, which results in low intestinal calcium absorption and thus PTH stimulation, would lead to a constant elevation of 1,25(OH)_2_D_3_.

**Figure 1:**
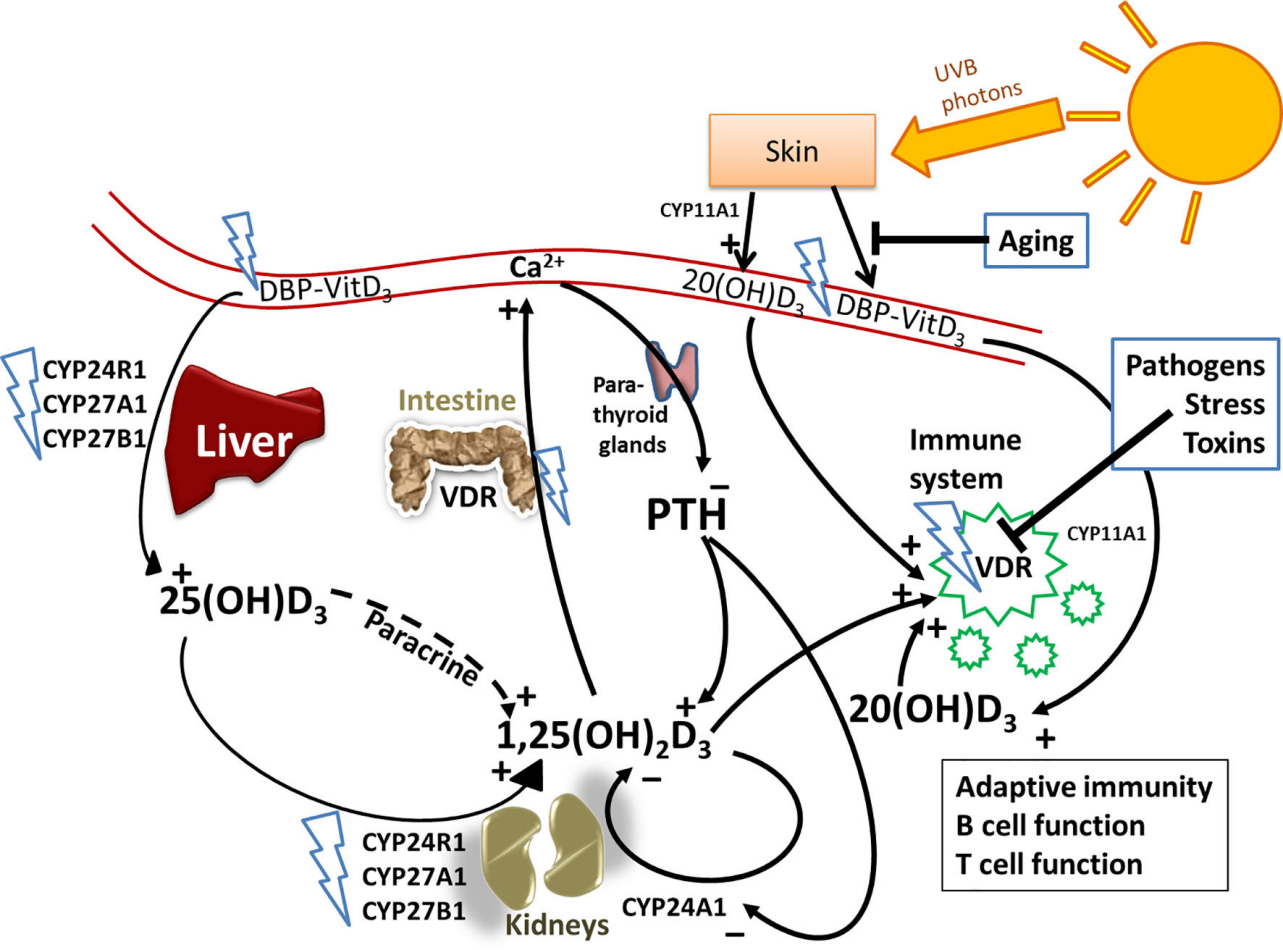
Vitamin D metabolism and the sites of vitamin D resistance acquirement. Vitamin D_3_ is mainly produced in the skin through solar ultraviolet-B (UVB) radiation and gets transported through blood by binding to vitamin D binding protein (DBP). The liver is the systemic production site of 25(OH)D_3_ *via* the enzymes CYP2R1, CYP27A1 or CYP27B1. 25(OH)D_3_ then gets converted to the active hormone 1,25(OH)_2_D_3_ by a second hydroxylation which occurs mainly in the kidneys or in other tissues through paracrine production. 1,25(OH)_2_D_3_ binds to the vitamin D receptor (VDR) to promote intestinal calcium (Ca) absorption and other Ca-mobilization pathways (e.g. in bone), so that ionized Ca levels in serum rise. The paraythroid glands sense Ca^2+^ levels, and secrete parathyroid hormone (PTH). PTH in turn promotes the production of 1,25(OH)_2_D_3_, e.g. by inhibiting CYP24A1 which catalyzes the first degradation step of 1,25(OH)_2_D_3_. Besides 1,25(OH)_2_D_3_, there are other vitamin D3-derived hydroxy-metabolites that exert hormone-like actions. For example, skin also expresses CYP11A1, which promotes the conversion of vitamin D_3_ into 20(OH)D_3_, which has non-calcemic biological effects, for example on immune cells. The blue flashes show where polymorphisms can interfere with normal vitamin D metabolism, building the basis for the development of acquired vitamin D resistance. Additional factors that impair vitamin D signaling are highlighted in blue boxes.

In case of a disturbed VDR response, e.g., caused by SNPs, an increased 1,25(OH)_2_D_3_ concentration is urgently needed to maintain the integrity of VDR signaling. Therefore, an elevated PTH concentration can result from vitamin D resistance mediated by dysfunctional VDR signaling. It follows that a decreased 25(OH)D_3_/1,25(OH)_2_D_3_ ratio could be perceived as a biomarker for vitamin D resistance. However, because PTH elevation is causally responsible for an increased 1,25(OH)_2_D_3_ level, PTH is a clinically sufficient sensitive surrogate biomarker for vitamin D resistance (**Figure 1**). Especially since, according to our experience, a disturbed 25(OH)D_3_/1,25(OH)_2_D_3_ ratio is perceived as not sufficiently reliable. A reason could be that some cases of vitamin D resistance are based on SNPs in enzymes catalyzing production of 1,25(OH)_2_D_3_ such as *CYP27B1*, as originally proposed by Coimbra and his co-workers (30). In this case, PTH would be elevated, but its action on expression of this dysfunctional enzyme is not sufficient to raise 1,25(OH)_2_D_3_ levels.

## Development of Vitamin D Resistance

As we have just illustrated, susceptibility for vitamin D resistance could arise from multiple polymorphisms of genes expressing for different proteins within the vitamin D system: the cytochrome-P450 enzymes (hydroxylases) needed for the conversion of vitamin D_2_/D_3_ into the activated hormone form (CYP2R1, CYP27A1 and CYP27B1), the DBP needed for vitamin D transport, the cell-surface receptor megalin-cubilin, which is the membrane receptor for the 1,25(OH)_2_D_3_/DBP complex, the VDR itself or the recently discovered other receptors for vitamin D hydroxy derivatives such as RORα and RORγ. Indeed, inactivating polymorphisms of the *CYP2R1* (31) or *CYP27B1* (32) genes, promoter polymorphisms of *CYP24A1* (33) or *RORA* (20), and SNPs of the *VDR* gene (34–38) have all been associated with autoimmune diseases. However, as suggested by research on hereditary vitamin D resistance, the VDR is probably the most vulnerable part of the vitamin D metabolic system and constitutes the most likely or at least most potent manifestation of acquired vitamin D resistance.

### The Vitamin D Receptor

The VDR is a steroid receptor expressed in almost all cell types of the human body, in particular most immune cells (38). The intracellular distribution of the VDR is usually 75-80% in the nucleus, 15-20% in the cytosol and 3-5% in the plasma membrane (39). Binding of 1,25(OH)_2_D_3_ induces translocation of cytosolic VDR into the nucleus in a dose-, time- and temperature-dependent manner (40). In the nucleus, the liganded VDR forms a heterodimer with the unliganded retinoid-X receptor which then binds to DNA and recruits either coactivator or corepressor proteins and other transcription factors to exert gene regulatory functions (39). Due to this property, the VDR is classified as a member of the nuclear receptor family. Other members of this “superfamily” of nuclear receptors are, among others, the thyroid receptor, the estrogen, progesterone and testosterone receptors, the retinoid-X receptor as well as the cortisol receptor (41). The high importance of the VDR is underscored by the great number of genes that it regulates, both as a classical transcription factor and through epigenetic effects (42). For example, Seuter et al. have shown that 1,25(OH)_2_D_3_ binding to the VDR modulated the transcription of 1204 genes in the human monocytic cell line THP-1 over 24 hours, mostly by modulating the accessibility to their respective chromatin regions (43). The 46 chromatin strands, which measure up to 5 cm in length and have a diameter of approximately 0.01 mm, are thereby modified in a way that opens up the target genes for reading (44). This epigenetic function of the VDR is exerted in almost all tissue types, which underlines its important role as a regulatory protein well beyond its known function in calcium metabolism (42, 43).

Critically, several *VDR* SNPs have been revealed that are increasingly associated with multiple autoimmune diseases, either individually or as haplotypes (34–38). For MS, e.g., a recent meta-analysis revealed a significant association with rs731236 (TaqI) polymorphisms when comparing heterozygous (Tt) with homozygous (TT) genotypes (45). The rs731236 GG genotype was also a significant predictor of low vitamin D responsiveness in a supplementation study of 100 Arab women, together with the rs7116978 (*CYP2R1* gene) CC genotype (46). This study also provided an overview showing that the rs7116978 G allele and rs7116978 C allele reached high population prevalence of up to 40% or 70%, respectively, depending on ethnicity (46). It is known that certain haplotypes of *VDR* polymorphisms can influence its mRNA expression levels, stability and protein translation efficiency (47). Furthermore, work of Booth et al. who studied polymorphisms within the context of VDR binding to genes in human monocytes and dendritic cells, suggests that certain polymorphisms predispose to autoimmune diseases by perturbing VDR binding at autoimmune disease risk gene variants (38). In other words, SNPs of the VDR may predispose to acquired vitamin D resistance, which may become manifest during aging and lead to the development of an autoimmune disease, if additional factors accumulate that contribute to an impairment of VDR signaling. These factors will be reviewed in the next subsection.

### The Vitamin D Receptor Is Regulated by Multiple Factors

With first evidence for a VDR in 550 million-year old boneless vertebrates, the VDR is a long-established regulatory protein (48). From an evolutionary perspective it appears plausible that such a conserved and seminal protein is subject to multiple influencing factors. We elaborate on this using the example of the glucocorticoids in relation to the VDR.

Glucocorticoids (cortisol, cortisone) are excreted as part of a stress reaction (fight or flight, psychological stress) and ensure the supply of energy needed to solve the problem at hand. Within this context, the inhibition of the VDR appears logical, since it would ensure the shift of energy from the immune to motor systems. However, if the VDR is chronically inhibited, e.g., upon long-term cortisone treatment or chronic stress, this would explain the occurrence of pathological consequences such as osteoporosis. The clinically important immune-suppressive effect of glucocorticoids could be explained in part by the VDR blockade. Also the positive immune modulatory effect of low-dose cortisol treatment could be due to an influence on the VDR.

The literature contains an increasing number of data confirming this hypothesis. For example, chronically elevated glucocorticoid levels have been shown to compromise the effects of 1,25(OH)_2_D_3_ and promote the development of osteoporosis (49). Administration of 10 mg prednisolone, an artificial glucocorticoid, for seven days to healthy males resulted in enhanced renal calcium excretion, an elevation of biomarkers of bone degeneration and an increase in PTH levels. These effects could be compensated by the administration of 1,25(OH)_2_D_3_ (50). Another study showed that dexamethasone, another synthetic glucocorticoid, is able to potentiate the VDR-mediated transcription of CYP24A1 by a mechanism involving the recruitment of the glucocorticoid receptor to the *CYP24A1* promoter region and its cooperation with C/EBPβ; since CYP24A1 catalizes the first step of 1,25(OH)_2_D_3_ degradation, dexamethasone effectively reduced 1,25(OH)_2_D_3_ concentrations (51). Finally, multiple putative glucocorticoid responsive elements could be identified within the *VDR* gene, which suggest a regulation of VDR expression through endogenous glucocorticoids (52). This is consistent with type II diabetics having both higher cortisol secretion (53) and lower VDR mRNA and protein levels (54) compared to healthy controls. In general, both inhibitory and activating tissue-specific effects on the VDR could be observed upon dexamethasone administration (52, 55).

Estrogens appear to positively stimulate vitamin D metabolism through a direct effect on the VDR (56, 57). An activation of the thyroid receptor, on the other hand, can have an inhibitory effect on vitamin D regulation *via* the VDR (58). These examples demonstrate that the response of the VDR can be modulated by a variety of physiological influences.

In addition, further pathophysiological mechanisms have emerged during the evolution of life. Due to the profound influence of vitamin D on the immune system it appears reasonable from an evolutionary standpoint to view the VDR as a strategically important target for pathological insults that aim to evade the immune system. For multiple tumor cell lines an anti-proliferative effect of 1,25(OH)_2_D_3_ has been described (34). An attenuation of these anti-proliferative effects would confer a growth advantage for tumor cells. Along these lines, colorectal cancer cells have been shown to regulate the expression or responsiveness of the VDR (59). A blockade of the VDR was also revealed as the pathophysiological mechanism exploited by osteosarcoma cells (60).

An increasing number of studies also describe the VDR as a strategic target of various pathogens. Lipopolysaccharides for example, which are sepsis inducing bacterial toxins, inhibit the expression of the VDR within THP-1 human monocytes (61).

In mammals, one of the central functions of the protein caspase-3 is the induction of programmed cell death (apoptosis). Thereby, it is possibly necessary to inhibit cellular vitamin D metabolism. Indeed, it was shown that caspase-3 is able to inhibit vitamin D metabolism by binding to and inactivating the VDR (62). Pathogens such as *A. salmonicida*, *Legionella pneumophila*, *E. coli* or *Yersinia* spp. in turn are able to interact with, or activate caspase-3 within cells, in this way inhibiting the VDR and attenuating the immune response of their host (62, 63).

Blockade of the VDR has also been described for other pathogens. A carefully conducted study has investigated the impact of *Borrelia burgdorferi* on the transcriptome of monocytes using whole genome microarrays [Supplementary Table S2 in (64)]. This technique revealed a 60-fold downregulation of the VDR by live *Borrelia*. In human B lymphocytes, it was shown that their infection with Epstein-Barr-virus (EBV) inhibits VDR mRNA and protein expression (65). Yenamandra et al. confirmed this VDR blockade through EBV in B lymphocytes (66) and later showed that the EBNA-3 protein is responsible for this blockade by its affinity to bind to the VDR (67). Finally, human cytomegalovirus induced a 88% inhibition of VDR expression in infected cells, leading to a gradual increase of the *CYP27B1* gene to 970%; notably, no downregulation of the VDR was observed for influenza or adenovirus infection (68). The mechanisms how pathogens disrupt vitamin D signaling may provide a missing link between the association of infections and autoimmune pathogenesis. Indeed, pathogen infections have been discussed as triggers of autoimmune diseases for a longer time (69). For example, infections with campylobacter, EBV or influenza viruses have been causally connected to the development of Guillain-Barré syndrome (70). Attention has also been paid to the link between EBV infection and MS development (71). As described above, blockade of the VDR by EBV could thereby provide a plausible underlying mechanism behind this link.

**Figure 2:**
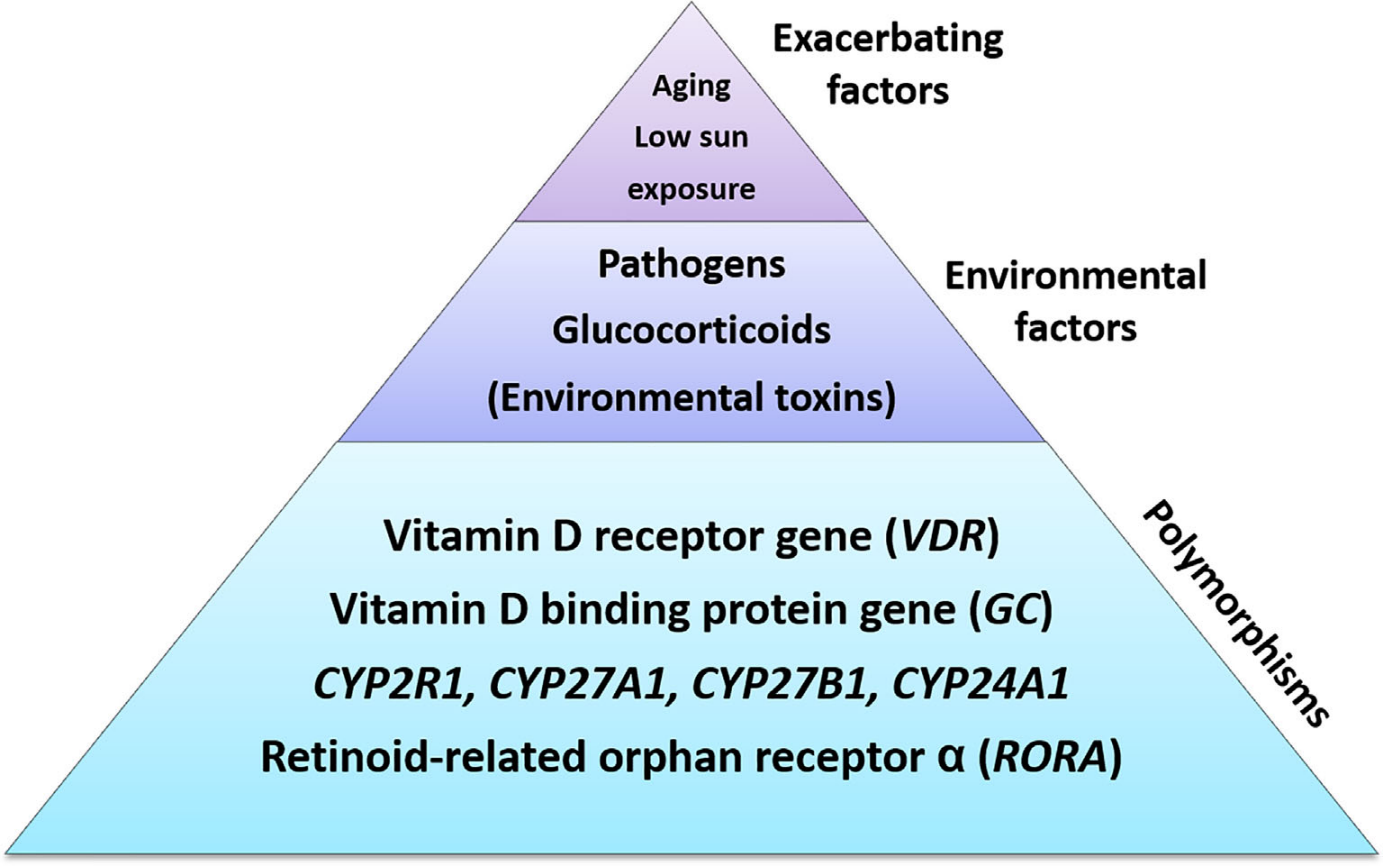
The etiology of acquired vitamin D resistance. Polymorphisms of genes within the vitamin D system constitute the basis for a susceptibitly towards developing vitamin D resistance, and hence autoimmune diseases. Partial blockades of the vitamin D receptor through pathogens, glucocorticoids (chronic stress) and – putatively – environmental toxins such as heavy metals may interact with such a susceptibility so that vitamin D resistance emerges. Finally, low sun exposure and aging, which correlate with autoimmune diseases as well, will exacerbate this situation further.

In summary, all these examples demonstrate a more or less potent influence on the vitamin D system *via* the VDR by physiological and pathophysiological influences. In particular, the pathogen-mediated temporary or chronic blockade of the VDR can be explained from an evolutionary view in which pathogens have developed ways to attack this key target in order to downregulate their host’s immune response. We thus propose the hypothesis that an interplay between acquired blockades of the VDR and polymorphisms affecting autoimmune disease susceptibility in either the *VDR* or other genes of the vitamin D system are able to cause a progressively severe form of low vitamin D responsiveness, which we refer to as acquired vitamin D resistance and which ultimately mediates the development of an autoimmune disease (**Figure 2**). Two other autoimmune disease-related factors fit nicely into this picture, namely a lack of sun exposure and the aging process, as intestinal vitamin D_3_ absorption (72), endogenous skin production (73) and hydroxylation (74) all have been shown to decrease with aging. Finally, environmental toxins could also be integrated into this hypothesis if they interfere with vitamin D metabolism. For example aluminum, which has been found in high concentrations in brain tissue from MS patients (75), was able to decrease renal CYP27B1 activity in the chick (76). Here, future research on the effects of environmental toxins such as aluminum and cadmium on the vitamin D system is urgently needed.

## Discussion

### Consequences of Vitamin D Resistance

The expression of the VDR as a gene regulatory protein in almost all tissues of the human body suggests functional roles well beyond those classically associated with calcium metabolism. Along these lines, vitamin D is more and more viewed as essential for maintaining physiological homeostasis. Vitamin D deficiency is associated with a plethora of cardiovascular and metabolic diseases including cancer, hypertension, infectious and autoimmune diseases (2, 7, 34). The proof of VDR expression in multiple cell types of the immune system such as CD4+/CD8+ lymphocytes, neutrophils and activated T-cell lymphocytes or antigen presenting cells (monocytes, macrophages or dendritic cells) underlines the importance of this receptor within this system (38). Mechanistically, multiple different effects of 1,25(OH)_2_D_3_ on the immune system have been described, including, but not limited to, chemotaxis, phagocytosis, proliferation, differentiation, cytokine production, antigen presentation, antibody, cathelicidin and hydrogen peroxide production and memory cell buildup (34). Only recently, Carlberg et al. identified a number of key genes of the immune system which are expressed by the VDR upon 1,25(OH)_2_D_3_ activation (48). Besides genes whose proteins play an essential role within the innate immune system by producing antimicrobial peptides, another group of proteins was identified which are closely connected to autoimmune processes. An example is the transmembrane protein Ninjurin1 (NINJ1) which is regulated by 1,25(OH)_2_D_3_. NINJ1 is involved in transmigration of antigen-presenting cells across the blood-brain barrier and in the pathogenesis of MS (77).

There also is an epigenetic influence of the VDR on genes of the immune system. An *in vivo* proof-of-principle study investigated chromatin accessibility in monocytes obtained from a human subject before and after a vitamin D bolus dose of 2000 µg (80,000 I.U.) (78). The bolus dose, which raised 25(OH)D_3_ levels by 7.6 ng/ml after two days, resulted in a significant opening or closing of hundreds of gene loci, the most prominent of which belonged to the human leucocyte antigen (HLA) system coded on chromosome 6. With the help of antigen-presenting proteins of the HLA system, the immune system is able to differentiate between endogenous and exogenous proteins. Therefore, a dysfunction within the HLA system predisposes for the development of autoimmune diseases. Polymorphisms of the HLA system for example constitute the most significant genetic influence on MS (79). Interestingly, a vitamin D responsive element could be identified at a gene within the HLA-DRB1 region, which is closely associated with the development of MS (80). Besides the epigenetic influence, this finding also underlines the transcriptional effect of vitamin D on the pathogenesis of this disease.

Looking beyond the box of autoimmune diseases to the mounting evidence of Vitamin D as a player in tumor development (81, 82), significant associations between SNPs of vitamin D system genes and common types of cancer (prostate, breast, colorectal and skin cancer) have been reported (83, 84), suggesting a similar predisposing role of SNPs for cancer as for autoimmune diseases. In these cases, a partial blockade of the VDR by pathogens could be another pathway within the established infectious etiology of cancer (85, 86).

In summary, there is much plausibility for acquired vitamin D resistance playing a pathological role in the development of autoimmune diseases, in this way providing an important component for our understanding and explanation of these diseases.

### Therapy of Vitamin D Resistance

Currently no causal and therefore reliable therapies exist for correcting a blockade of the VDR. The only effective therapy to date is the high-dose vitamin D protocol, known after its inventor as the Coimbra protocol (30). With up to approximately 1000 I.U. vitamin D_3_ per kg body weight, the highest therapeutic vitamin D starting doses are thereby administered for the treatment of MS. For other autoimmune diseases much lower doses appear sufficient (**Table 1**). Hypercalcemia may be a major concern raised against this protocol. However, as discussed in more detail below, the vitamin D resistance appears to confer an intrinsic protection against hypercalcemia. In addition, the therapy requires the patient to take some precautionary measures. Besides the avoidance of milk products and a minimum fluid intake of 2.5 l/day, patients will need to consistently monitor multiple blood parameters and undergo regular sonographic checks of their kidneys. Practically, this requires close and regular contact with a certified Coimbra practitioner.

**Table 1:** Initial vitamin D_3_ doses used within the Coimbra protocol for the treatment of autoimmune diseases.

Initial vitamin D_3_ dose [I.U./kg body weight]	Disease
**1,000**	Multiple sclerosis
**300-500**	Rheumatoid arthritis, Systemic lupus erythematosus, Psoriatic arthritis, Psoriasis, Crohn’s disease, Ulcerative colitis
**300**	Systemic scleroderma, Ankylosing spondylitis, Hashimoto’s thyroiditis
**150**	Others

Doses are successively adapted (almost always lowered) during follow-up according to a standardized procedure which considers parathyroid hormone and calcium concentrations as well as the clinical condition of the patient.

The Coimbra protocol follows a long medical tradition in which receptor resistances are compensated by applying higher doses. This is exemplified using a simplified analogy of insulin resistance therapy:

The current practice in type 2 diabetes management consists of calculating the required external insulin dose according to blood glucose levels. Thereby it is almost irrelevant how high the injected insulin dose actually is. It is the correction of the target parameter, i.e. blood glucose, which is pivotal. To achieve this goal, total daily insulin doses exceeding 100 I.U. can be utilized (87). These are doses that would normally be toxic in the absence of an underlying insulin resistance. The diabetic patient is to some extent protected against severe side effects of what would usually be considered an overdose, because this dose only acts physiologically – provided the applied doses are calculated correctly and there is close supervision by a physician.

**Figure 3:**
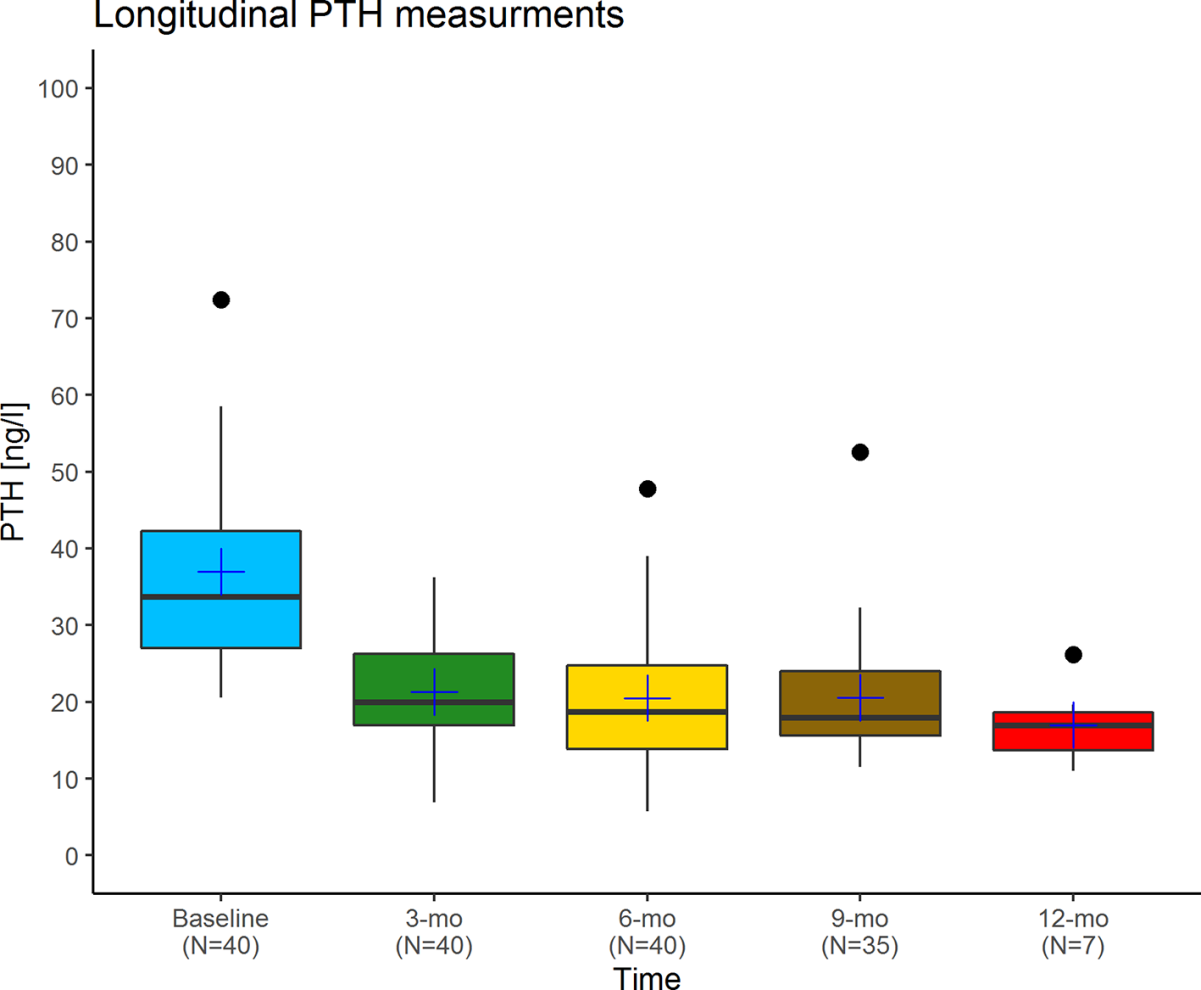
Longitudinal PTH measurements in a cohort of 41 relapsing-remitting multiple sclerosis patients treated by the corresponding author (DL) with the Coimbra protocol. Black lines indicate the median, blue crosses the mean. At three months follow-up, PTH levels had dropped significantly compared to the baseline measurement (Wilcoxon rank sum test, p<0.001), while there were no significant differences between any of the follow-up measurements. The reduction of PTH concentrations into the middle of the lower third of its laboratory-specific reference range is judged as an indicator for overcoming vitamin D resistance.

Although we acknowledge that the causes of insulin resistance are very different from those of vitamin D resistance, and high-dose insulin injections cause comparatively more side effects than high-dose vitamin D_3_ injections, the above analogy illustrates the principles of the Coimbra protocol: The calculation of the required vitamin D dose depends on the grade of vitamin D resistance and the stage of the particular disease and incorporates the PTH concentration in analogy to the insulin/blood glucose example. Provided close supervision by an experienced physician, the applied high doses of vitamin D_3_ are causing neither hypercalcemia nor kidney damage. They will only exert physiological effects – in this way, the underlying vitamin D resistance acts protective against what would normally be considered a potentially toxic dose. In **Supplementary Table 1** we provide data collected in the corresponding author’s practice (DL) revealing no case of calcium excursion in a cohort of 41 patients observed over at least six months. The development of their PTH concentrations is plotted in **Figure 3** and shows a significant decline at three months follow-up.

However, one case of a 39-year old symptomatic MS patient with hypercalcemia and renal insufficiency has been reported by a Swiss team of physicians (88). After seven months on the Coimbra protocol with 100,000 I.U. of vitamin D_3_ daily, his calcium concentration at hospital admission was 3.0 mmol/L, his 25(OH)D_3_ level 697 ng/ml (1742 nmol/l), and his PTH was 96 ng/l. The PTH level was thus much higher than typically observed on the Coimbra protocol (**Figure 3**). A possible explanation is that the patient was a carrier of the Multiple Endocrine Neoplasia, type 1 (MEN1) gene and had a history of elevated PTH levels already prior to starting the Coimbra protocol. Although this case highlights the possibility of high-dose vitamin D toxicity within the Coimbra protocol, it appears to be very rare. However, it also highlights the need for a diagnostic assessment of hyperparathyroidism prior to initiating the protocol as well as a regular endocrinological monitoring during therapy. It is noteworthy that the patient described in the case report wanted the authors to provide a brief account of his own standpoint according to which he and other patients he knew had improved their MS symptoms significantly on the protocol (88).

Such subjective improvements of autoimmune disease patients as well as safety data have also been collected within the network of Coimbra protocol physicians in Germany; these will be subject to future publications.

Finally, we would briefly like to mention the opinion held by some therapists that vitamin D administration is counterindicated in patients with vitamin D resistance. This hypothesis is inconsistent with the medical experience and understanding according to which resistances can be compensated with normal-to-high doses as described above. In particular, it is inconsistent with the experience gained by many Coimbra protocol physicians specialized in high dose vitamin D therapy that have collected many treatment successes thus far.

In summary, we have reviewed evidence for the hypothesis of an acquired form of vitamin D resistance, developing on the basis of a genetic susceptibility from certain SNPs within the vitamin D system and its interplay with chronic stress and/or pathogen infections that are able to partially block the VDR. Other factors that have been associated with autoimmune diseases such as low sun exposure, aging or environmental toxins could easily be integrated into this hypothesis since they would further exacerbate developing vitamin D resistance arising from the described mechanisms (**Figure 2**).

The hypothesis of acquired vitamin D resistance thus provides a plausible pathomechanism for the development of autoimmune diseases. We consider its therapeutic exploitation by high-dose vitamin D administration as a promising approach. Our key messages reflecting the knowledge about vitamin D resistance and its treatment are summarized in **Table 2**.

**Table 2:** Key points discussed in this paper.

Vitamin D therapy and resistance - update 2021
Latest research in the field of individual vitamin D responsiveness undermines the empirical concept and clinical experience of high dose vitamin D_3_ therapy in autoimmune disease.
Polymorphisms within genes of the vitamin D system could make an individual susceptible for developing low vitamin D responsiveness, with acquired vitamin D resistance as an extreme form exacerbated by other factors causing blockades of the vitamin D receptor.
There is no linear or exponential relationship between vitamin D and calcium blood levels.
Hypercalcemia is not a first line risk of high dose vitamin D_3_ therapy.
Parathyroid hormone level in serum remains the key information for Vitamin D_3_ dose finding concepts in autoimmune disease as long as there is no better information about individual factors determining vitamin D responsiveness available.
Based on these insights high dose vitamin D_3_ therapy should be included into the therapeutic measures of all autoimmune diseases on an individual basis only.
Careful planning and controlling of high dose vitamin D_3_ therapy by qualified therapists is mandatory.
The high dose vitamin D_3_ concept in autoimmune disease should be transferred to other areas of vitamin D_3_ therapy – above all cancer, but also diabetes and cardiovascular diseases to name but a few.

## Data Availability Statement

The original contributions presented in the study are included in the article/**Supplementary Material**, further inquiries can be directed to the corresponding authors.

## Author Contributions

DL and RJK drafted the initial manuscript. DL and BS collected the data. FS and JS edited the manuscript. All authors contributed to the article and approved the submitted version.

## Conflict of Interest

DL and BS are certified Coimbra practitioners. BS owns the practice that apply the here described Coimbra protocol to patients with autoimmune diseases.

The remaining authors declare that the research was conducted in the absense of any commercial or financial relationships that could be construed as a potential conflict of interest.
