# Supplementary material for: Vitamin D Resistance as a Possible Cause of Autoimmune Diseases: A Hypothesis Confirmed by a Therapeutic High-Dose Vitamin D Protocol
Source: Front Immunol. 2021 Apr 7;12:655739. doi: 10.3389/fimmu.2021.655739 (PMC8058406; doi:10.3389/fimmu.2021.655739)
Supplement: Supplementary file 1 [file Table_1.docx]

**Supplementary Table 1**: Characteristics and PTH development of 41 patients with relapsing-remitting multiple sclerosis .

| **ID** | **Gender** | **Age** | **Baseline 25(OH)D_3_**  **[ng/ml]** | **Baseline**  **PTH**  **[ng/l]** | **3-month**  **PTH**  **[ng/l]** | **6-month**  **PTH**  **[ng/l]** | **9-month**  **PTH**  **[ng/l]** | **12-month**  **PTH**  **[ng/l]** | **Baseline Ca**  **[mmol/l]** | **Last follow-up Ca**  **[mmol/L]** |
| --- | --- | --- | --- | --- | --- | --- | --- | --- | --- | --- |
| 1 | f | 28 | 21.6 | 33.1 | 17.7 | 14.7 | 11.5 | 17.0 | 2.38 | 2.2 |
| 2 | f | 52 | 23.2 | 24.5 | 18.5 | 27.9 | 21.5 | 17.7 | 2.3 | 2.46 |
| 3 | f | 45 | 37.7 | 21.7 | 14.5 | 19.5 | 22.0 |  | 2.27 | 2.36 |
| 4 | f | 48 | 38.8 | 58.5 | 24.9 | 22.3 | 17.9 |  | 2.09 | 2.25 |
| 5 | m | 28 | 8.4 | 32.0 |  |  |  | 11.0 | 2.53 | 2.5 |
| 6 | f | 33 | 17.2 | 72.4 | 26.0 | 35.0 | 52.6 | 26.2 | 2.38 | 2.33 |
| 7 | f | 29 | 19.6 | 26.0 | 23.0 | 17.0 | 19.0 |  | 2.15 | 2.19 |
| 8 | f | 39 | - | 27.0 | 20.0 | 15.0 | 18.0 |  | 2.26 | 2.28 |
| 9 | m | 29 | 40.0 | 23.0 | 20.0 | 17.0 | 18.0 |  | 2.52 | 2.52 |
| 10 | f | 20 | 18.8 | 52.9 | 24.1 | 34.7 | 27.1 |  | 2.35 | 2.34 |
| 11 | f | 29 | 16.4 | 24.3 | 16.2 | 14.4 | 17.2 | 19.6 | 2.23 | 2.3 |
| 12 | f | 33 | 23.8 | 20.6 | 19.3 | 15.6 | 17.4 |  | 2.37 | 2.3 |
| 13 | m | 27 | 39.1 | 29.2 | 30.1 | 12.2 | 27.3 |  | 2.32 | 2.35 |
| 14 | f | 52 | 18.8 | 41.7 | 27.2 | 11.8 | 15.7 |  | 2.51 | 2.42 |
| 15 | f | 33 | 50.8 | 41.0 | 19.0 | 24.0 | 23.0 |  | 2.35 | 2.34 |
| 16 | f | 56 | 15.3 | 37.7 | 21.0 | 12.0 | 14.1 |  | 2.33 | 2.38 |
| 17 | f | 23 | 15.0 | 28.0 | 11.0 | 12.0 | 14.0 |  | 2.39 | 2.46 |
| 18 | m | 26 | 16.4 | 37.7 | 22.6 | 19.8 | 17.0 |  | 2.37 | 2.39 |
| 19 | m | 31 | 11.4 | 54.0 | 32.0 | 28.0 | 25.0 |  | 2.41 | 2.45 |
| 20 | m | 27 | - | 23.6 | 13.4 | 22.6 | 16.4 | 14.5 | 2.38 | 2.32 |
| 21 | m | 39 | 12.5 | 33.3 | 31.8 | 12.1 | 26.6 |  | 2.39 | 2.3 |
| 22 | f | 42 | 11.2 | 24.1 | 10.0 | 10.7 | 14.6 | 12.9 | 2.34 | 2.45 |
| 23 | m | 37 | 80.0 | 27.0 | 17.0 | 22.0 | 14.0 |  | 2.49 | 2.4 |
| 24 | f | 39 | - | - | 6.9 | 5.7 | 11.8 |  | 2.33 | 2.39 |
| 25 | f | 34 | 14.1 | 48.7 | 22.0 | 30.4 | 15.5 |  | 2.4 | 2.4 |
| 26 | f | 33 | 14.8 | 21.8 | 16.9 | 23.3 |  |  | 2.3 | 2.33 |
| 27 | f | 48 | 10.4 | 39.0 | 36.0 | 39.0 | 32.0 |  | 2.51 | 2.45 |
| 28 | m | 47 | 9.8 | 36.1 | 16.2 | 13.4 |  |  | 2.38 | 2.5 |
| 29 | m | 38 | 5.9 | 41.5 | 19.0 | 19.7 | 20.9 |  | 2.4 | 2.4 |
| 30 | f | 25 | 13.6 | 32.2 | 27.0 | 32.8 | 16.5 |  | 2.4 | 2.3 |
| 31 | f | 35 | 11.0 | 55.9 | 36.2 | 18.0 |  |  | 2.46 | 2.49 |
| 32 | f | 45 | 30.0 | 34.2 | 30.0 | 20.0 | 21.7 |  | 2.42 | 2.36 |
| 33 | f | 18 | 33.6 | 36.0 | 15.0 | 11.0 | 14.0 |  | 2.41 | 2.38 |
| 34 | f | 30 | 12.8 | 57.0 | 13.0 | 17.0 | 28.0 |  | 2.17 | 2.24 |
| 35 | f | 31 | - | 29.2 | 21.7 | 19.8 | 18.9 |  | 2.43 | 2.5 |
| 36 | f | 27 | 12.8 | 30.1 | 22.6 | 17.9 |  |  | 2.2 | 2.5 |
| 37 | f | 21 | 7.2 | 57.9 | 18.6 | 30.7 |  |  | 2.34 | 2.33 |
| 38 | f | 49 | 24.8 | 28.2 | 17.9 | 12.2 | 18.8 |  | 2.33 | 2.32 |
| 39 | m | 52 | 5.4 | 44.0 | 18.0 | 14.0 | 14.0 |  | 2.25 | 2.37 |
| 40 | f | 44 | 40.2 | 53.7 | 27.8 | 47.8 | 32.3 |  | 2.18 | 2.2 |
| 41 | f | 49 | 17.5 | 41.0 | 28.0 | 27.0 | 25.0 |  | 2.34 | 2.44 |

The last follow-up calcium concentration was measured at the time of last PTH concentration measurement that is reported in the table. f: Female; m: Male
